# ﻿*Elaphomycescastilloi* (Elaphomycetaceae, Ascomycota) and *Entolomasecotioides* (Entolomataceae, Basidiomycota), two new sequestrate fungi from tropical montane cloud forest from south Mexico

**DOI:** 10.3897/mycokeys.96.98320

**Published:** 2023-04-03

**Authors:** Javier Isaac de la Fuente, Jesús García-Jiménez, Tania Raymundo, Marcos Sánchez-Flores, Ricardo Valenzuela, Gonzalo Guevara-Guerrero, Erika Cecilia Pérez-Ovando, César Ramiro Martínez-González

**Affiliations:** 1 Colegio de Postgraduados, Km 36.5 Montecillo, Texcoco, 56230, Estado de México, Mexico Colegio de Postgraduados Texcoco Mexico; 2 Tecnológico Nacional de México, Instituto Tecnológico de Ciudad Victoria, Blvd. Emilio Portes Gil #1301Pte, 87010, Ciudad Victoria, Tamaulipas, Mexico Instituto Tecnológico de Ciudad Victoria Ciudad Victoria Mexico; 3 Instituto Politécnico Nacional, Escuela Nacional de Ciencias Biológicas, Departamento de Botánica, Laboratorio de Micología, 11340, Ciudad de Mexico, Mexico Instituto Politécnico Nacional Ciudad de Mexico Mexico; 4 Instituto de ciencias biólogicas, Universidad de Ciencias y Artes de Chiapas, Libramiento Norte Poniente, 29039, Tuxtla Gutiérrez, Chiapas, Mexico Universidad de Ciencias y Artes de Chiapas Tuxtla Gutiérrez Mexico

**Keywords:** Chiapas, hypogeous fungi, mycorrhizal fungi, phylogeny, truffle-like fungi

## Abstract

Two new species of sequestrate fungi are described from south Mexico based on morphological and molecular evidences. Here we describe *Elaphomycescastilloi* characterized by the yellowish mycelial mat, dull blue gleba and ascospores of 9.7–11.5 µm; *Entolomasecotioides* is characterized by the secotioid basidiomata, sulcate, pale cream pileus, and basidiospores of 7–13 × 5–9 µm. Both species grow in montane cloud forest under *Quercus* sp. in the state of Chiapas, Mexico. Descriptions, photographs, and multilocus phylogeny for both species are presented.

## ﻿Introduction

Sequestrate fungi are characterized by producing hypogeous sporome, protected by a thick peridium to avoid desiccation, changes in temperature, and humidity (Thiers 1984). Due to this morphological modification, these species are not capable of dispersing their spores through the air, so they use other strategies such as producing aromas to attract animals that consume and disperse them through their feces (Castellano et al. 1986; Caiafa et al. 2021). Many species are ectomycorrhizal and are associated with the roots of angiosperms and gymnosperms, mainly trees and shrubs of the genera *Abies*, *Coccoloba*, *Dycimbe*, *Eucalyptus*, *Quercus*, and *Pinus* (Trappe et al. 2009). Some saprobic species are also known, mainly from tropical forests (de la Fuente et al. 2021). This diversity in the tropics is just beginning to be discovered and, according to Sulzbacher et al. (2016), there are more than 1,500 described species in the world with more being regularly added.

Hypogeous fungi have been studied in Mexico since the 1970’s and the studies of García-Romero et al. (1970) and Trappe and Guzmán (1971), although the first record of a hypogeous fungus was *Melanogasterumbriniglebus* Trappe & Guzmán, recorded in Chihuahua by Lumbholz in the late 1800’s (Cázares et al. 2008). Since the 2000’s, species have been regularly described, mainly species from temperate forests. Approximately 100 species of hypogeous fungi are currently known, mainly from northeastern Mexico and the Neovolcanic axis, areas where more studies have been carried out (Trappe and Guzmán 1971; Cázares et al. 1992; Guevara-Guerrero et al. 2014; Gómez-Reyes et al. 2018);however, many Mexican states and different types of vegetation have been under-explored.

The state of Chiapas is located in southern Mexico and is an important reservoir of montane cloud forest (Williams-Linera 1991). This type of vegetation represents less than 1% of the national coverage and is declining alarmingly (SEMARNAT 2010). This kind of forest is located between 1500 to 2500 m above sea level. Annual precipitation exceeds 1500 mm. The most common trees in this forest type include *Pinus*, *Quercus*, *Lyquidambar* and *Magnolia* (González-Espinoza et al. 2012). Due to several species of fungi growth associated with the roots of trees, it is possible to carry out successful reforestation experiments with native species. Hence knowing the native fungi of the forest becomes a priority in their conservation (Martínez-Reyes et al. 2012). In this study, two new species of hypogeous fungi are described: *Elaphomycescastilloi*, characterized by the blackish ascoma covered with yellow mycelial mat and bluish gleba, and *Entolomasecotioides*, characterized by its pale-colored secotioid basidiome. Both species grow in montane cloud forest under *Quercus* species. Photographs, descriptions and multiloci phylogeny are presented for both species.

## ﻿Materials and methods

### ﻿Sampling data

**Figure 1.**
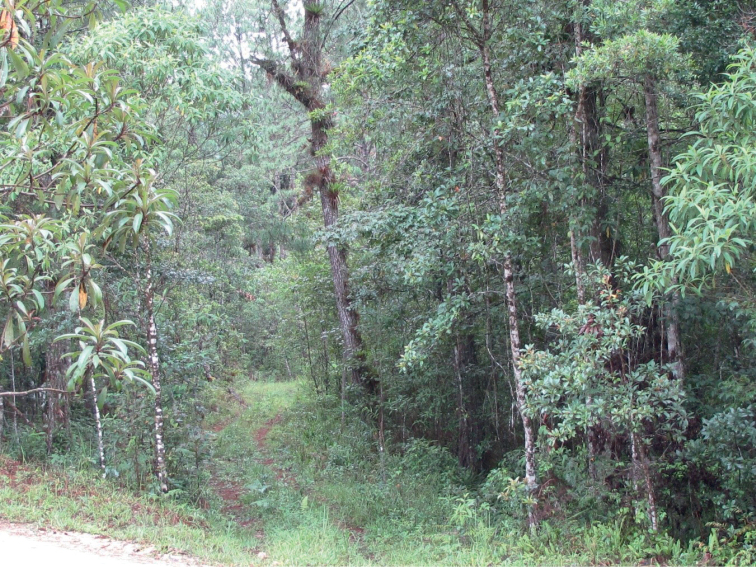
Montane cloud forest at La Trinitaria, Chiapas, Mexico.

Mycological explorations were carried out in the state of Chiapas, southern Mexico (Fig. 1). The dominant vegetation in the sampling site corresponds to a tropical montane cloud forest. For the collection of the specimens, the protocols proposed by Castellano et al. (1986) were followed. The specimens were registered and herborized. A color chart was used for color terminology (Kornerup and Wanscher 1978). Hand cuts were made on dried specimens and temporal preparation was mounted in order to observe microstructures. KOH 5% and Melzer’s reagent were used to observe amyloid reactions. At least 30 spores and other microstructures were measured using an optical microscope (Motic ba310, San Antonio, USA) to obtain the average length (L), average width (W) and Q ratio (Q). The scanning electron microscope (Hitachi Su 1510, Hitachi, Japan) of IB-UNAM (Mexico City, Mexico) was utilized to observe spore ornamentation. The collected specimens were deposited in ITCV.

### ﻿DNA extraction, amplification, and sequencing

The DNA was obtained from herbarium specimens (Tables 1, 2). The CTAB protocol of Martínez-González et al. (2017) was used to extract genomic DNA. The DNA was quantified with a Nanodrop 2000c (Thermo ScientificTM, Wilmington, USA). We prepared dilutions from each sample at 20 ng/µL to amplify the Internal Transcribed Spacer rDNA-ITS1 5.8S rDNA-ITS2 (ITS), the nuclear large subunit ribosomal DNA (LSU) and the second largest subunit of the RNA polymerase II gene (rpb2). The reaction mixture for PCRs was performed on a final volume of 15 µL containing 1× buffer, 0.8 mM dNTPs mix, 20 pmol of each primer, 2 units of GoTaq DNA (Promega, USA) and 100 ng of template DNA. The PCR products were verified by agarose gel electrophoresis. The gels were run for 1 h at 95 V cm^−3^ in 1.5% agarose and 1× TAE buffer (Tris Acetate-EDTA). The gel was stained with GelRed (Biotium, USA) and the bands were visualized in an Infinity 3000 transilluminator (Vilber Lourmat, Eberhardzell, Germany). The amplified products were purified with the ExoSAP Purification kit (Affymetrix, USA), following the manufacturer’s instructions. They were quantified and prepared for the sequence reaction using a BigDye Terminator v.3.1 (Applied Biosystems, USA). These products were sequenced in both directions with an Applied Biosystem model 3730XL (Applied BioSystems, Foster City, USA), at the Instituto de Biología of the Universidad Nacional Autónoma de México (UNAM). The sequences obtained were compared with the original chromatograms to detect and correct possible reading errors. The sequences of both strands of each of the genes were analyzed, edited and assembled using the BioEdit v. 7.0.5 (Hall 1999) to generate a consensus sequence which compared with those deposited in GenBank using the tool BLASTN v. 2.2.9 (Zhang et al. 2000).

**Table 1.** GenbBank accession numbers corresponding to the sequences used in the phylogenetic analyses for *Elaphomycescastilloi*. In bold the accessions of the new species.

Species name	Isolate/Voucher/strain	Locality	GenBank Accessions	
			ITS	nrLSU
*Elaphomycesaculeatus*	16952	Italy	JF907985	–
*Elaphomycesadamizans*	TH9660 (Type)	Guyana	KT694133	KT694144
Elaphomycesaff.decipiens	GO-2009-211	Mexico	KC152093	–
***Elaphomycescastilloi***	**García 18640 (Holotype)**	**Mexico**	**OP821418**	**OP824738**
	**Guevara 1162 (Paratype)**	**Mexico**	**OP821419**	**OP824739**
*Elaphomycescitrinus*	16955	Spain	JF907986	–
	LIP0001141	Spain	–	KX238822
*Elaphomycescompleximurus*	TH8880	Guyana	JN711441	–
	TH8880	Guyana	NR121522	–
*Elaphomycesdecipiens*	Trappe 12436	USA	EU837229	–
	Trappe 28269	USA	EU846311	–
*Elaphomycesdigitatus*	MCA1923	Guyana	–	JN713148
*Elaphomycesfavosus*	TH10015	Cameroon	KT694134	KT694145
	TH9859 (type)	Cameroon	KT694138	KY694149
	TH9897	Cameroon	KT694136	KT694146
*Elaphomycesgranulatus*	KM47712	UK	EU784197	–
*Elaphomycesguangdongensis*	KT-TW09-030	Taiwan	HM357249	–
	KT-TW09-031	Taiwan	HM357250	HM357248
*Elaphomycesiupperticellus*	TH9934	Cameroon	KT694141	KT694142
	THDJA 39 (type)	Cameroon	KT694139	KT694143
*Elaphomyceslabryinthinus*	TH9918 (type)	Cameroon	KT694137	KT694148
*Elaphomycesleveillei*	16960	Italy	JF907987	–
*Elaphomycesmaculatus*	16961	Italy	JF907988	–
*Elaphomycesmuricatus*	Hy14	Finland	GU550112	–
	HA38	Latvia	KR019869	–
*Elaphomyces* sp.	HB1	Indonesia	–	LC010285
	YM144	Japan	–	AB848482
	AM3GA3A4	USA	–	JQ272414
	LM5570B	Hungary	–	KM576391
	73812	UK	–	FJ876187
	GM1332	USA	–	KF359559
Uncultured *Elaphomyces*	141A	Canada	KM403019	KM403019

**Table 2.** GenbBank accession numbers corresponding to the sequences used in the phylogenetic analyses for *Entolomasecotioides*. The accessions of the new species are in bold.

Species name	Isolate/Voucher/strain	GenBank Accessions		
		ITS	nrLSU	*rpb2*
Entolomaaff.prunuloides	628	–	–	KC710159
Entolomaaff.sinuatum	TRTC156542	JN021020	–	–
*Entolomaalbidum*	620	KC710102	KC710151	–
*Entolomaalbomagnum*	427	KC710065	KC710137	–
*Entolomaaraneosum*	14	GQ289153	GQ289255	GQ289293
*Entolomaasterosporum*	TENN064538	JF706309	–	JF706312
*Entolomabaronii*	L644	KC710093	–	–
*Entolomacaccabus*	17	KC710063	GQ289155	GQ289227
*Entolomacaesiolamellatum*	626	KC710126	KC710157	–
*Entolomacallidermum*	512	KC710115	KC710153	–
***Entolomasecotioides***	**García 18817 (Holotype)**	**OP821420**	**OP824740**	**KC265752**
	**Guevara 1173 (Paratype)**	**OP821421**	**OP824741**	KC265753
*Entolomacf.griseoluridum*	LNM221111	KC710118	–	–
*Entolomachilense*	MES 1012	KY462399	–	–
*Entolomaclypeatum*	41	KC710059	KC710136	–
*Entolomacoeruleogracilis*	216	KC710069	–	–
*Entolomaconferendum*	30	KC710055	KC710133	KC710191
*Entolomacorneri*	607	KC710058	KC710135	–
*Entolomacretaceum*	2010039	KC710090	–	–
*Entolomaflavifolium*	621	KC710097	KC710150	–
*Entolomafumosobrunneum*	MEN 2005113	KC710124	KC710155	–
*Entolomagracilior*	2011043	KC710079	–	–
*Entolomahypogaeum*	K382	NR119416	–	AB692019
*Entolomakermandii*	222	–	GQ289173	GQ289244
*Entolomalividoalbum*	233	KC710114	KC710152	–
*Entolomaluridum*	2005108	KC710091	KC710146	KC710192
*Entolomamadidum*	221	KC710127	KC710158	–
	67195	KC710130	–	–
*Entolomamanganaense*	215	KC710085	KC710143	–
*Entolomamyrmecophilum*	231	KC710120	–	–
*Entolomaochreoprunuloides*	15721	KC710111	–	–
	632	KC710092	KC710147	–
Entolomaochreoprunuloidesf.hyacinthinum	6	KC710105	–	–
*Entolomaperbloxamii*	2010037	KC710095	–	–
*Entolomaprismaticum*	K381	AB691998	–	AB692016
*Entolomaprunuloides*	40	KC710073	GQ289184	GQ289255
*Entolomapseudoprunuloides*	627	KC710078	KC710140	–
*Entolomasequestratum*	MFLU 12-2045	MH323431	MT344186	MT349886
*Entolomasinuatum*	182	KC710116	KC710154	–
*Entolomasordidulum*	1	KC710062	GQ289194	GQ289265
*Entolomasphagneti*	209	KC710061	GQ289195	–
*Entolomasubsinuatum*	YL2269	KC710096	KC710149	–
*Entolomatrachyosporum*	405	KC710088	GQ289198	–
*Entolomaturbidum*	27	KC710060	GQ289201	GQ289269
*Entolomawhiteae*	629	KC710084	KC710142	–
*Entolomaalcedicolor*	210	KC710123	GQ289152	GQ289224
*Entocybenitidum*	24	KC710122	GQ289175	GQ289246

### ﻿Phylogenetic analyses

To explore the phylogenetic relationships of the new species of *Elaphomyces*, an alignment was made based on the taxonomic sampling employed by Paz et al. (2017). Outgroup was selected according to Paz et al. (2017). Each gene region was independently aligned using the online version of MAFFT v. 7 (Katoh et al. 2002, 2017; Katoh and Standley 2013). Alignment was reviewed in PhyDE v.10.0 (Müller et al. 2005), followed by minor manual adjustments to ensure character homology between taxa. The matrix was formed for ITS by 24 taxa (697 characters), while LSU by 19 taxa (845 characters). The aligned matrices were concatenated into a single matrix (32 taxa, 1542 characters). Two partitioning schemes were established: one for the ITS and one for the LSU, which were established using the option to minimize the stop codon with Mesquite v3.70 (Maddison and Maddison 2017).

To explore the phylogenetic relationships of the new species of *Entoloma*, an alignment was made based on the taxonomic sampling employed by Elliott et al. (2020). The outgroup was selected according to Elliott et al. (2020). Each gene region was independently aligned using the online version of MAFFT v. 7 (Katoh et al. 2002, 2017; Katoh and Standley 2013). Alignment was reviewed in PhyDE v.10.0 (Müller et al. 2005), followed by minor manual adjustments to ensure character homology between taxa. The matrix was formed for ITS by 45 taxa (700 characters), for LSU by 31 taxa (831 characters), while rpb2 consisted of 17 taxa (670 characters). The aligned matrices were concatenated into a single matrix (47 taxa, 2201 characters). Five partitioning schemes were established: one for the ITS, one for the LSU and three for rpb2 gene region, which were established using the option to minimize the stop codon with Mesquite v3.70 (Maddison and Maddison 2017).

Phylogenetic inferences were estimated with maximum likelihood (ML) in RAxML v. 8.2.10 (Stamatakis 2014) with a GTR + G model of nucleotide substitution. To assess branch support, 10,000 nonparametric rapid bootstrap pseudoreplicates were run with the GTRCAT model. For Bayesian posterior probability (PP), the best evolutionary model for alignment was sought using Partition Finder (Frandsen et al. 2015; Lanfear et al. 2014, 2017). Phylogeny analyses was performed using MrBayes v. 3.2.6 ×64 (Huelsenbeck and Ronquist 2001). The information block for the matrix includes two simultaneous runs, four Montecarlo chains, temperature set to 0.2 and sampling 10 million generations (standard deviation ≤ 0.1) with trees sampled every 1000 generations. The first 25% of samples were discarded as burn-in, and stationarity was checked in Tracer v. 1.6 (Rambaut et al. 2014). Trees were visualized and optimized in FigTree v. 1.4.4 (Rambaut 2014), and then edited in Adobe Illustrator vCS4 (Adobe Systems, Inc., San Jose, CA).

## ﻿Results

### ﻿Phylogenetic analyses

**Figure 2.**
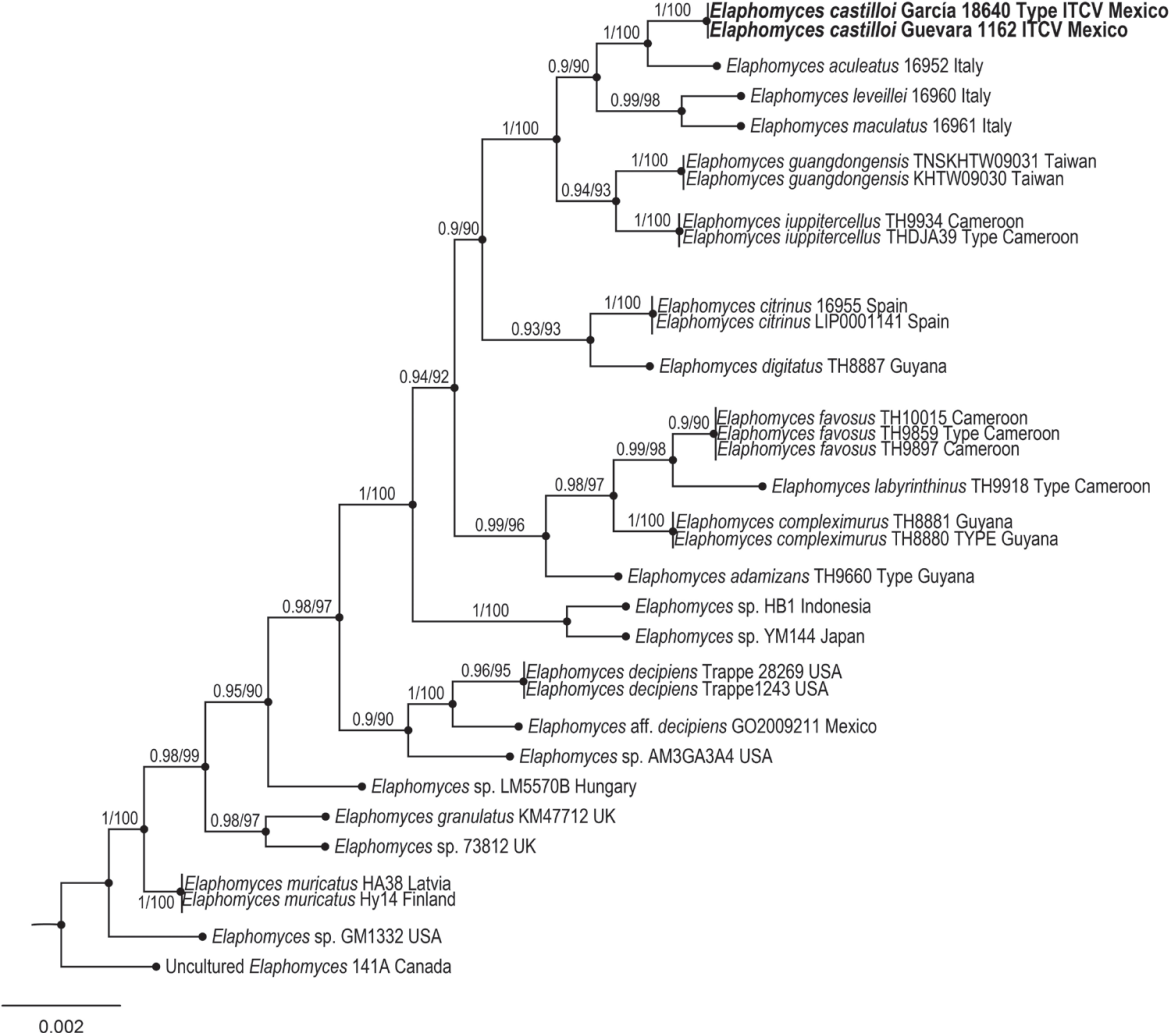
Bayesian inference phylogram of ITS-LSU sequences data for *Elaphomycescastilloi*. Posterior probability (left of slash) from Bayesian analysis and Bootstrap support (right of slash).

**Figure 3.**
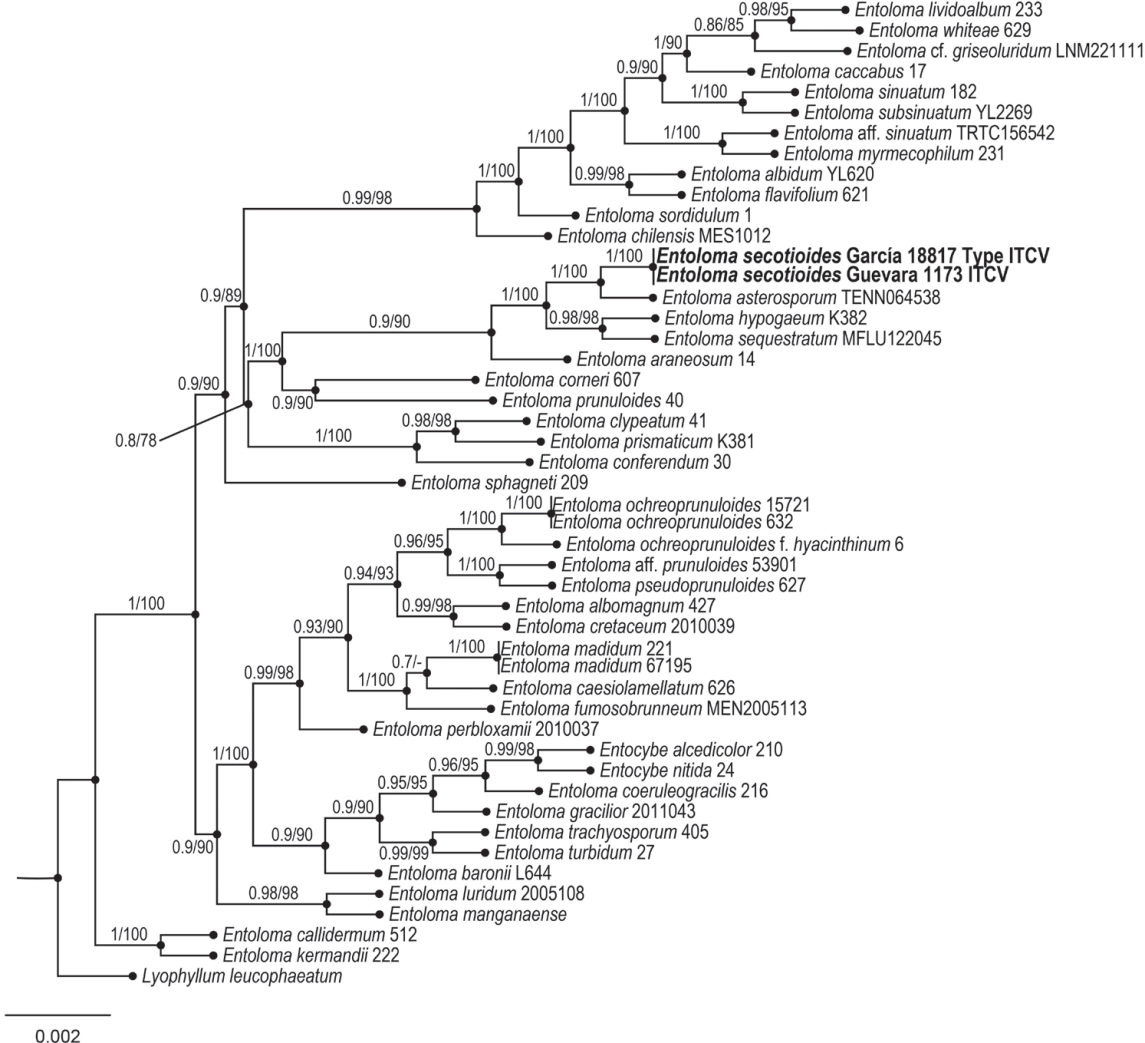
Bayesian inference phylogram of ITS-LSU-RPB2 sequences for *Entolomasecotioides*. Posterior probability (left of slash) from Bayesian analysis and Bootstrap support (right of slash).

The ITS and LSU sequences obtained from *Elaphomycescastilloi* and ITS, LSU and rpb2 from *Entolomasecotioides* were deposited in GenBank. The two simultaneous Bayesian runs continued until the convergence parameters were met, and the standard deviation fell below 0.001 after 10 million generations for *Elaphomycescastilloi* and 0.002 for *Entolomasecotioides*. No significant changes in tree topology trace or cumulative split frequencies of selected nodes were observed after about 0.33 million generations for *E.castilloi* and 0.45 million generations for *E.secotioides*, so the first 2,500,000 sampled trees (25%) were discarded as burn-in. Both the Bayesian analyses and Maximum Likelihood (Figs 2, 3) recovered *Elaphomycescastilloi* supporting the existence of one new taxon distinctive from related species of *Elaphomyces* (1 Bayesian Posterior Probability and 100% bootstrap proportion for Maximum Likelihood) and *Entolomasecotioides*, supporting the existence of one new taxon distinctive from related species of *Entoloma* (1 Bayesian Posterior Probability and 100% bootstrap proportion for Maximum Likelihood).

### ﻿Taxonomy

#### ﻿ Elaphomyces castilloi

Taxon classificationFungiEurotialesElaphomycetaceae

J. García, Guevara & de la Fuente sp. nov.

2EA9F762-8AF7-5D77-B4EC-CCC2FFE368FE

MB842037

. Fig. 4A–G

##### Type material.

***Holotype*.** Mexico. Chiapas: la Trinitaria Municipality, Lagunas de Monte bello, alt. 1004 m, 16°53'N, 93°27'W, 16 August 2019, J. García 18640 (Holotype-ITCV).

##### Diagnosis.

*Elaphomycescastilloi* differs from other species of the genus by the following combination of characteristics: ascomata embedded in a yellow mycelial mat, dull blue powdery gleba, and globose reticulate ascospores (9.7–11.5 µm).

##### Etymology.

The species was named *castilloi* in honor of José Castillo Tovar (*ad memoriam*), a Mexican pioneer mycologist dedicated to studying the fungi from northeast Mexico.

##### Description.

***Ascomata*** globose to ellipsoid, 14–32 mm, embedded in a thick, yellowish orange (4A7) to deep yellow (4A8), with a membranous mycelial mat, occasionally incorporating soil particles, and debris, loose but compacted near the peridium, easily detachable. ***Peridium*** surface black, slightly rough, carbonaceous, inner peridium grayish brown (8D3), sometimes with white mycelial strand, near the gleba forming a discontinuous layer. ***Gleba*** powdery, dull blue (23D5), compacted when young, becoming loose when mature, with scattered grey hyphae (25C1); odor and taste fungoid.

***Mycelial mat hyphae*** cylindrical, 2–6 µm diameter, septate, hyaline, thin-walled, loosely arranged. ***Epicutis***: 125–200 µm diameter, composed of compacted hyphae, 3–8 µm diameter, strongly interwoven, subglobose to irregular, black in 5% KOH, thick-walled. ***Subcutis*** 500–650 µm diameter, composed by prostrated and compacted hyphae, 8–15 µm in diameter, hyaline to dull grey in 5% KOH (25D4), becoming irregular near the gleba, thin-walled. ***Asci*** subglobose, 32–38 × 25.8–30.1 µm, 5 to 8-spored, hyaline, thin-walled. ***Ascospores*** 9.7–11.5 µm (n = 30), globose, rarely subglobose, reticulated, projecting up to 1.9–2.7 µm, forming small bridges (less than 2 µm), with obtuse tips, golden brown color (5D7), thick-walled.

##### Additional material examined.

Mexico. Chiapas: la Trinitaria Municipality, Lagunas de Monte bello, alt. 1004 m, 16°53'N, 93°27'W, 16 August 2011, Guevara 1102 (Paratype-ITCV). ITS: OP821419, LSU: OP824739.

##### Distribution.

Known only from the Mexican state of Chiapas, growing scattered, and hypogeous under *Quercus* sp. in montane cloud forest.

##### Notes.

*Elaphomycescastilloi* is phylogenetically close to *Elaphomycesaculeatus* Vittad. from Italy, the last one with similar ascospore color and ornamentation. It was previously reported from Mexico by Gómez-Reyes et al. (2012). *Elaphomycesaculeatus* has a reddish peridium and dark-brown gleba; meanwhile, *E.castilloi* has dark peridium and bluish gleba. The yellow mycelial mat and the small ascospores resemble those of *Elaphomycescitrinus* Vittad. (Section Malacodermei). However, it differs by the smaller ascocarp (less than 10 mm), the brownish peridium in young specimens, and by its geographic distribution (Europe) (Pegler et al. 1993). Although the morphological features of the new species are typical in the *Malacodermei*, these are also seldom observed in the *Ceratogaster* (Paz et al. 2017).

**Figure 4.**
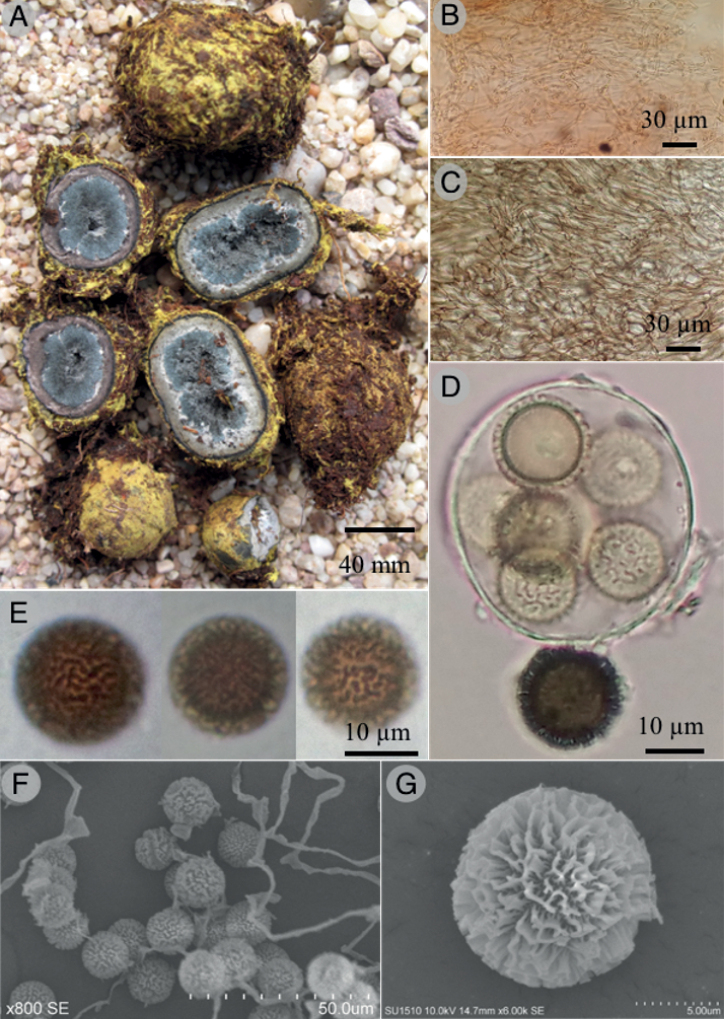
*Elaphomycescastilloi* (Holotype) **A** ascomata **B** mycelial mat hyphae **C** subcutis **D** asci **E, F** ascospores in KOH **G** detail of ascospore ornamentation in SEM.

#### ﻿ Entoloma secotioides

Taxon classificationFungiAgaricalesEntolomataceae

J. García, Guevara & de la Fuente sp. nov.

37665240-C4DC-5FE4-B489-54BB74543689

MB842038

. Fig. 5A–F

##### Type material.

***Holotype*.** Mexico. Chiapas: la Trinitaria Municipality, Lagunas de Monte bello, alt. 1004 m, 16°53'N, 93°27'W, 16 August 2019, J. García 18817 (Holotype-ITCV).

##### Diagnosis.

*Entolomasecotioides* is characterized by cream colored, sulcate, secotioid basidiomata, not anastomosed gills, and angular basidiospores (7–13 × 5–9 µm).

##### Etymology.

Named *secotioides* due to the secotioid basidiomata.

##### Description.

Pileus 12–15 mm, subglobose, flattened when young, becoming depressed when mature, sulcate, pale yellow (4A3) to light yellow (4A5), slightly velvety, margin incurved enclosing the hymenium, dry in appearance, sometimes with brownish fibrils. Hymenophore lamellate, slightly irregular, pale orange to orange white (5A2) to light yellow (4A5), not exposed even in mature specimens. Stipe 4–9 × 3–4 mm, cylindrical or absent, light yellow (4A5), smooth or finely fibrillose. Taste and odor fungoid, mild.

**Figure 5.**
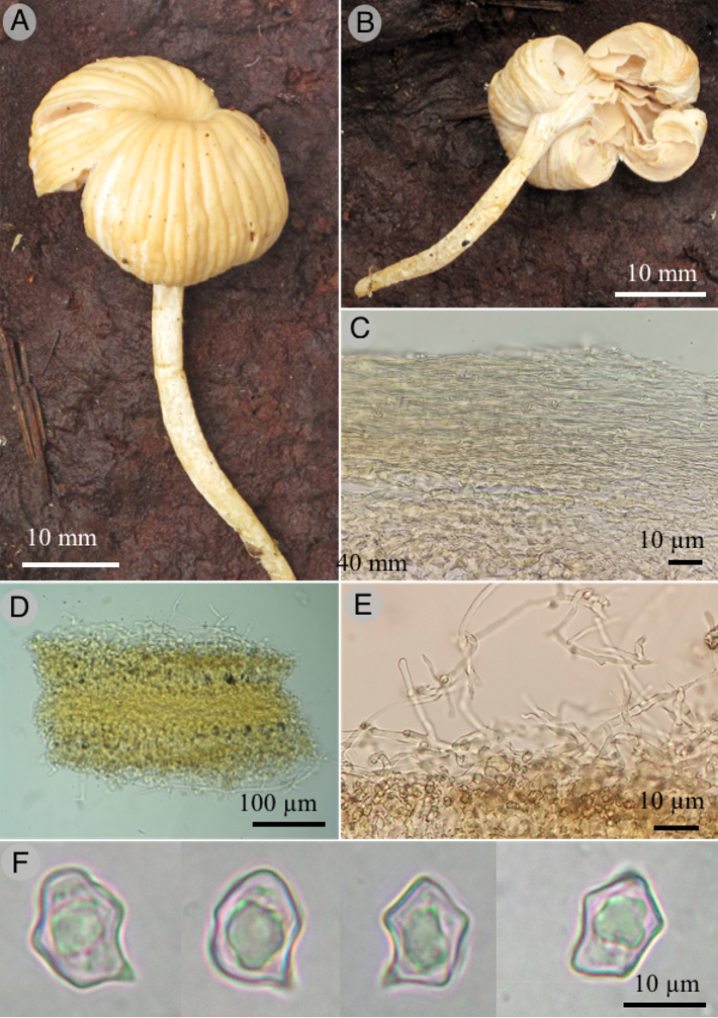
*Entolomasecotioides* (Holotype) **A, B** basidiomata showing the pileus, hymenia, and stipe **C** peridium **D, E** details of the hymenium **F** basidiospores in KOH.

***Peridium*** 70–300 µm composed of loosely interwoven or horizontally arranged hyphae, 4–7 µm in diameter, septate, bifurcate, hyaline to pastel green in 5% KOH (27A4), not reacting with Melzer, with clavate terminal cells, thin-walled. ***Hymenophoral trama*** 45–110 µm in diameter, composed of interwoven compacted hyphae, 4–9 µm in diameter, light orange in 5% KOH (5A4), thin-walled. ***Basidia*** 27–35 × 5–10 µm forming palisades, clavate, hyaline, thin-walled, embedded by a layer of loosely interwoven hyphae which arise from the trama, 6–11 µm diameter, sometimes branched, inflate at the septum, sometimes with terminal cells cystidioid or cylindrical, thin-walled. ***Basidiospores*** 7–13 × 5–9 µm, (L = 10.2, W = 7.1, Q = 1–2.2, n = 30), angular, rare nodulose, with 6–8 sides, some with conspicuous hilar appendix up to 3 µm, hyaline to pastel green (27A4), not reacting with Melzer reagent, smooth, thin-walled.

##### Distribution.

Known only from the state of Chiapas, growing sub hypogeous under *Quercus* sp. and *Pinus* sp. in montane cloud forest.

##### Additional material studied.

Mexico. Chiapas: la Trinitaria Municipality, Lagunas de Monte bello, alt. 1004 m, 16°53'N, 93°27'W, 16 August 2019, Guevara 1173 (Paratype-ITCV). ITS: OP821421; LSU: OP824741; RPB2: KC265753.

##### Notes.

*Entolomasecotioides* is characterized by pale-cream basidiomata, enclosed, not anastomosed gills, and angular basidiospores 7–13 × 5–9 µm. *Entolomacalongei* (E. Horak & G. Moreno) Noordel. & Co-David has gray-brown pileus, loculate gleba, and basidiospores 6–10 µm (Horak and Moreno 1998); *Entolomachilensis* (E. Horak) Noordel. & Co-David also has grayish pileus, loculate gleba, and basidiospores 9–11 × 6.5–7.5 µm (Horak 1963). Both species differ from *E.secotioides* mainly in the basidiomata color (pale-cream *vs.* grayish-brown) and hymenophore shape (slightly irregular *vs.* locules). The new species is phylogenetically close to *E.asterosporum* (Coker & Couch) T.J. Baroni & Matheny, differing from *E.secotioides* by having the globose sporome, pungent odor and smell, and larger spores (up to 16 µm) (Baroni and Matheny 2011).

## ﻿Discussion

Hypogeous fungi in Mexico have been scarcely studied compared to epigeous fungi; however, from the 2000s, new species have been regularly described, mainly from temperate forests (Guevara-Guerrero et al. 2014; Gómez-Reyes et al. 2018). In the case of *Elaphomyces*, it is one of the best represented genera in the country because it is associated with a large number of hosts, mainly *Pinus* and *Quercus* (Trappe and Guzmán 1971; Cázares et al. 1992; Castellano et al. 2012; Gómez-Reyes et al. 2012). Some species of *Elaphomyces* have even been described as culturally important. *Elaphomycesmuricatus* has been reported for ritual or medicinal use (Trappe et al. 1979). For *Entolomasecotioides*, this is the first record of a sequestrate *Entoloma* in Mexico, these being mostly of the previously recorded species pileate-stipitate. Although the species has been described growing under *Quercus* species, there are no data on its ecological habits and these are presented here as putatively associated with *Quercus*.

Chiapas is one of the states with the greatest biological richness in Mexico, only surpassed by Oaxaca (López-Guzmán et al. 2017). The diversity of fungi in this state spans approximately 850 species. Recent research suggests a fungal diversity between 20,000 and 49,000 species (Chanona-Gómez et al. 2007; Ruan-Soto et al. 2013; Kong et al. 2018). Efforts are currently being made to document this diversity, which is threatened by land use change. *Elaphomycescastilloi* represents the first record of the genus *Elaphomyces* in Chiapas and represents the southernmost distribution of the genus in Mexico. Another species reported in southern Mexico is *E.maculatus*, which has been reported in north Oaxaca in oak forests (Trappe et al. 1979).

The sequestrate fungi have been studied mostly in the temperate regions of the north and center of the country; *Elaphomycescastilloi* and *Entolomasecotioides* are new contributions that represent the first findings of sequestrate fungi from the montane cloud forest in Chiapas, more than 50%; of which have unfortunately disappeared; montane cloud forest constitutes less than 1% of the Mexican territory. However, it is vital to carry out samplings that include taxa from this ecosystem considering that its losses are so high. Some localities are deemed critical for conservation of this ecosystem which is considered “endangered” under the definition of the Official Mexican Law (SEMARNAT 2010). The degradation of the montane cloud forests in Chiapas is high, therefore the level of threat to the habitat of the new two species is also high. So far, they are only known from the type locality and it is necessary to increase the sampling to assess the current status of the species. Keeping the taxonomical studies about the fungi from the montane cloud forest could help to encourage its conservation and management.

## Supplementary Material

XML Treatment for Elaphomyces castilloi

XML Treatment for Entoloma secotioides
